# EGb761 Provides a Protective Effect against Aβ_1-42_ Oligomer-Induced Cell Damage and Blood-Brain Barrier Disruption in an *In Vitro* bEnd.3 Endothelial Model

**DOI:** 10.1371/journal.pone.0113126

**Published:** 2014-11-26

**Authors:** Wen-bin Wan, Lan Cao, Lu-mei Liu, Bill Kalionis, Chuan Chen, Xian-tao Tai, Ya-ming Li, Shi-jin Xia

**Affiliations:** 1 Department of Neurology, Zhongshan Hospital, Fudan University, Shanghai, China; 2 State Key Laboratory of Medical Neurobiology, School of Basic Medical Sciences, Fudan University, Shanghai, China; 3 Geriatrics Department of Traditional Chinese Medicine, Huadong Hospital, Fudan University, Shanghai, China; 4 Department of Perinatal Medicine Pregnancy Research Centre and University of Melbourne Department of Obstetrics and Gynaecology, Royal Women’s Hospital, Parkville, VIC, Australia; 5 Shanghai Geriatric Institute of Chinese Medicine, Shanghai, China; 6 School of Acupuncture, Massage and Rehabilitation, Yunnan University of Traditional Chinese Medicine, Kunming, China; 7 Shanghai Institute of Geriatrics, Huadong Hospital, Fudan University, Shanghai, China; University of Maryland School of Pharmacy, United States of America

## Abstract

Alzheimer’s disease (AD) is the most common form of senile dementia which is characterized by abnormal amyloid beta (Aβ) accumulation and deposition in brain parenchyma and cerebral capillaries, and leads to blood-brain barrier (BBB) disruption. Despite great progress in understanding the etiology of AD, the underlying pathogenic mechanism of BBB damage is still unclear, and no effective treatment has been devised. The standard *Ginkgo biloba* extract EGb761 has been widely used as a potential cognitive enhancer for the treatment of AD. However, the cellular mechanism underlying the effect remain to be clarified. In this study, we employed an immortalized endothelial cell line (bEnd.3) and incubation of Aβ_1–42_ oligomer, to mimic a monolayer BBB model under conditions found in the AD brain. We investigated the effect of EGb761 on BBB and found that Aβ_1–42_ oligomer-induced cell injury, apoptosis, and generation of intracellular reactive oxygen species (ROS), were attenuated by treatment with EGb761. Moreover, treatment of the cells with EGb761 decreased BBB permeability and increased tight junction scaffold protein levels including ZO-1, Claudin-5 and Occludin. We also found that the Aβ_1–42_ oligomer-induced upregulation of the receptor for advanced glycation end-products (RAGE), which mediates Aβ cytotoxicity and plays an essential role in AD progression, was significantly decreased by treatment with EGb761. To our knowledge, we provide the first direct *in vitro* evidence of an effect of EGb761 on the brain endothelium exposed to Aβ_1–42_ oligomer, and on the expression of tight junction (TJ) scaffold proteins and RAGE. Our results provide a new insight into a possible mechanism of action of EGb761. This study provides a rational basis for the therapeutic application of EGb761 in the treatment of AD.

## Introduction

*Ginkgo biloba* leaves are a type of medicinal herb and their extract has been shown to have neuroprotective properties and enhance cognitive functions [1], [2]. EGb761 is the standardized extract of *Ginkgo biloba* produced by Dr. Willar Schwabe Pharmaceuticals, which contains 22–27% flavonol glycosides, 5.4–6.6% terpene trilactones, 2.8–3.4% ginkgolides (A, B and C), 2.6–3.2% bilobalide, and less than 5 ppm ginkgolic acids [1]. Recently, EGb761 has received significant attention as a potential cognitive enhancer for the treatment of Alzheimer’s disease (AD) [1]–[4]. Substantial clinical and preclinical evidence indicates that EGb761 limits vascular and neural damage and has many beneficial effects that support its use in treating AD individuals [5]–[7]. However, the cellular and molecular mechanisms underlying these effects remain to be elucidated.

AD is the most common neurodegenerative disease that causes progressive cognitive and behavioral deterioration in the elderly [8], [9]. Extracellular deposition of the amyloid beta (Aβ) is widely accepted as an important event in the pathogenesis of AD [10], [11]. Aβ is considered to be one of the most acute neurotoxins in the central nervous system [10]–[12]. Very recently, cerebrovascular changes leading to blood-brain barrier (BBB) leakiness have been associated with Aβ deposition in the brains of AD individuals, and this may be involved in AD progression [13]–[15]. Despite great progress in understanding the etiology of AD, the process of deposition of Aβ aggregates in cerebral capillaries and the brain is still poorly understood and the underlying pathogenic mechanisms of BBB leakage remain unclear. Furthermore, no effective treatment has been devised.

The receptor for advanced glycation end-products (RAGE) is an essential transmembrane cell-signaling receptor, which binds free Aβ and mediates pathophysiological cellular responses, including oxidative stress, neurodegeneration, transport of circulating plasma Aβ across the BBB into the brain, and brain endothelial cell (EC) damage [16]–[19]. RAGE expression is increased in cells of the neurovascular unit in the brains of AD individuals, and in disease models of AD both *in vivo* and *in vitro* [19], [20]. This is particularly the case in models associated with an Aβ-rich environment [21]. More importantly, antagonizing RAGE expression, or RAGE-knockout studies, show that blocking the RAGE-Aβ interaction at the BBB suppresses the accumulation of Aβ in brain parenchyma [22], prevents Aβ-induced BBB disruption and ameliorates tight junction (TJ) scaffold protein expression [20]. These data suggest that RAGE is related to Aβ accumulation as well as disruption of BBB integrity, and that RAGE might be a potential therapeutic target for AD.

Recently, an *in vitro* study in a cell monolayer BBB model reported that EGb761 diminished cell injury induced by chronic hypoxia and hypoglycemia (CHH), and significantly reversed CHH-induced upregulation of RAGE expression [23]. Considering the protective properties of EGb761 and its therapeutic potential, we speculated that EGb761 treatment might have a protective effect on Aβ-induced BBB disruption by inhibition of RAGE. To testify our hypothesis, we employed an *in vitro* BBB model comprising an immortalized mouse brain capillary endothelial cell line (bEnd.3). Our study assessed the effects of Aβ_1–42_ oligomer treatment of bEnd.3 endothelial cells with respect to changes in the expression of RAGE, and TJ scaffold proteins including ZO-1, Claudin-5 and Occludin. Finally, we investigated the effect of EGb761 on Aβ_1–42_ oligomer treatment of bEnd.3 endothelial cells.

## Materials and Methods

### Reagents and antibodies

Lyophilized human Aβ_1–42_, purified by HPLC, was purchased from GL Biochem (Shanghai, China). EGb761 powder, a standardized *Ginkgo biloba* extract that contains two major active constituents 24% flavonol glycosides and 6% terpene trilactones, was purchased from Dr. Willmar Schwabe (Karlsruhe, Germany). The rabbit anti-ZO-1, anti-Claudin-5 and anti-Occludin antibodies were purchased from Invitrogen (CA, USA), whilst the rabbit anti-RAGE antibody was purchased from Millipore (MA, USA). The rabbit anti-GAPDH antibody was purchased from Santa Cruz Biotechnology (CA, USA) and the IRDye 680LT goat anti-rabbit IgG was purchased from LI-COR (CA, USA). MTT [3-(4, 5-dimethylthiazol-2-yl)-2, 5-diphenyl tetrazolium bromide] was purchased from Sigma (CA, USA). Sodium fluorescein (Na-F, MW: 376 Da) powder was purchased from Kayon Bio-tech Co.(Shanghai, China).

### Reagents preparation

Lyophilized human Aβ_1–42_ was used to prepare Aβ_1–42_ oligomer as described previously [24], [25]. Aβ_1–42_ was initially dissolved to 1 mM in hexafluoroisopropanol (HFIP, Sigma, USA) and aliquoted into sterile microcentrifuge tubes. Then, HFIP was removed under vacuum in a Speed Vac, and the peptide stored at −20°C. For oligomer preparation, 2 mM Aβ_1–42_ peptide that dissolved in dry dimethyl sulfoxide (DMSO, Sigma, USA) was subsequently diluted into ice-cold Opti-MEM (Gibco, USA) to bring the peptide to a final concentration of 100 µM. The solution was vortexed for 30 seconds, centrifuged for 1 minute, and incubated at 4°C for 24 h before use. EGb761 was dissolved in DMSO at a concentration of 200 mg/ml and stored at room temperature. The required concentrations of EGb761 were made by further dilution of the concentrated stock solution with Opti-MEM.

### Cell culture and treatments

Murine brain capillary endothelial cells (bEnd.3 obtained from ATCC, USA) were cultured in Dulbecco’s modified Eagle’s medium (DMEM, Gibco, USA) containing 4.5 g/l glucose supplemented with 10% fetal bovine serum (Gibco, USA), 100 U/ml penicillin and 100 µg/ml streptomycin (Gibco, USA) at 37°C in a humidified atmosphere containing 5% CO_2_ and subcultured every 3 days.

Cells were grown to 70–80% confluence prior to treatment. Before the treatments were applied, cells were rinsed in PBS and then the medium was replaced with Opti-MEM (Invitrogen, USA). For treatment of the cells exposed to Aβ_1–42_ oligomer and EGb761, the cells were pretreated with EGb761 for 2 h and then treated with Aβ_1–42_ oligomer.

### Measurement of cell viability

Cell viability was measured the using MTT assay. bEnd.3 cells were seeded onto 96-well plates and treated with EGb761 at different concentrations. MTT (20 µL of a 5 mg/ml stock, diluted in PBS) was added to each cell culture well containing 100 µL of medium. After 4 h incubation at 37°C, the medium was gently aspirated. Deposited formazan crystals were lysed in 100 µL DMSO by gently shaking the plate. Absorbance at 570 nm was measured using a micro plate reader (Bio-Rad). The cell viability (%) was expressed as a percentage relative to the untreated control cells.

### Detection of cell apoptosis

Apoptosis was observed by Hoechst-33258 staining (Apoptosis Hoechst staining kit; Beyotime, China). Briefly, cells were fixed in 0.5 mL of methanol for 15 min, followed by two washes with PBS. Cells were stained with 1 µg/mL Hoechst 33258 in a dark chamber at room temperature for 10 min and again washed twice in PBS. Cells were analyzed by fluorescence microscopy using excitation at 350 nm and emission at 460 nm. Apoptotic cells were identified on the basis of nuclear morphology changes such as chromatin condensation and fragmentation. In each group, ten fields of view were selected randomly and counted.

### Detection of intracellular ROS

The level of intracellular reactive oxygen species (ROS) was quantified using the Reactive Oxygen Species Assay Kit (Beyotime, China). DCFH-DA is oxidized by reactive oxygen species in viable cells to 2′,7′-dichlorofluorescein (DCF) which is highly fluorescent at 530 nm. Cells were washed three times with PBS and then DCFH-DA, diluted to a final concentration of 10 µM, was added and the cells were incubated for 30 min at 37°C in the dark. After washing three times with PBS, the stained cells in the 6-well plate were analyzed by inverted fluorescence microscopy (CKX41, OLYMPUS, Japan). The relative levels of fluorescence in cells were quantified by a multi-detection microplate reader (Bio-Rad) with excitation at 488 nm and emission at 525 nm. The level of intracellular ROS was expressed as the percentage of the control cells.

### BBB permeability assay

Transendothelial permeability was measured using Na-F as described previously [26], [27] with the following modifications. bEnd.3 cells (5×10^4^ cells/cm^2^) were cultured in the apical compartment, on a 0.4 µm pore size, 6.5 mm diameter polycarbonate membrane Transwell permeable insert (Corning). After the cells achieved confluence, 1.5 ml HHBS assay buffer (136 mM NaCl, 0.9 mM CaCl_2_, 0.5 mM MgCl_2_, 2.7 mM KCl, 1.5 mM KH_2_PO_4_, 10 mM Na H_2_PO_4_, 25 mM glucose, and 10 mM HEPES, pH 7.4) was added to the basolateral compartment. Culture medium in the apical compartment was replaced by 0.5 ml HHBS assay buffer containing 10 µg/ml Na-F (Kayon Biology). After 30 min, the medium from the basolatera compartments was removed and fluorescence in this medium was determined by a multiwell plate reader (Bio-Rad) at the wavelengths of 485 nm (excitation) and 535 nm (emission).

### Western blotting

Cells extracts were prepared by washing cells twice with PBS and resuspending in RIPA buffer (150 mM NaCl, 1% NP-40, 0.5% Sodium Deoxycholate, 0.1% SDS, 50 mM Tris-HCl (pH 7.4), 20 mM NaF, 20 mM EGTA, 1 mM DTT, 1 mM Na_3_VO_4_) with PMSF containing protease and phosphatase inhibitors. The extracts were then subjected to ultrasonication. Western blotting was performed to measure the change in tight junction protein levels including ZO-1, Claudin-5 and Occludin, and RAGE. Protein samples (30 µg total protein per lane) were subjected to 10% SDS-PAGE. After electrophoresis, protein was transferred onto a nitrocellulose (NC) blotting membrane (Millipore). Membranes were blocked with 5% fat-free milk for 1 h at room temperature, and then incubated overnight at 4°C with the following rabbit primary antibodies diluted to 1∶1000; anti-ZO-1 (Invitrogen, USA), anti-Claudin-5 (Invitrogen, USA), anti-Occludin (Invitrogen, USA), anti-RAGE (Millipore, USA) and anti-GAPDH (Santa Cruz, USA). Secondary goat anti-rabbit antibody (LI-COR, USA) was incubated with the filters for 1 h at room temperature. The images were captured using Odyssey infrared fluorescence imaging system (LI-COR, USA).

### Statistical analysis

All results are expressed as the mean ± S.E.M. Statistical analysis was performed using GraphPad Prism 5.0 software (GraphPad Software, Inc.). All experiments were repeated three times independently. Statistical significance of differences among different groups was analyzed by one-way analysis of variance (ANOVA) or student *t* test. A *p-*value<0.05 was considered statistically significant.

## Results

### EGb761 diminished Aβ_1-42_ oligomer-induced cell injury of bEnd.3 cells

**Figure 1:**
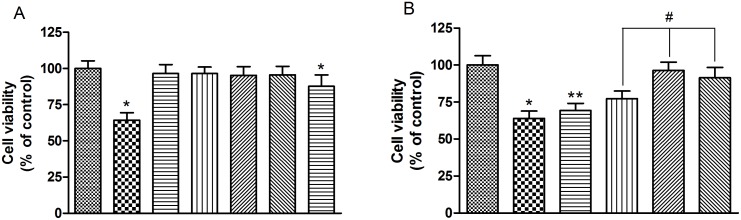
EGb761 increased cell viability against Aβ_1__–42_ oligomer toxicity. Panel A, bEnd.3 cells were incubated with various concentrations of EGb761 for 24 h and then cell viability was analyzed according to MTT assay by detecting the absorbance at 490 nm. Panel B, cells were incubated with or without various concentrations of EGb761 for 2 h, followed by incubation with 10 µM Aβ_1–42_ oligomer for 24 h. Subsequently, cell viability was determined by MTT assay. Results are shown as the Mean±S.E.M. (*p<0.01, Aβ versus Control; #p<0.01, EGb761+Aβ versus Aβ; **p<0.05, versus Aβ).

In this study, we first investigated whether EGb761 influenced the cell viability of bEnd.3 cells by MTT analysis. The results showed that incubation with various concentrations of EGb761 (25–200 µg/ml) in Opti-MEM did not lead to any significant changes in cell viability (**Fig. 1A**). However, at a concentration of 300 µg/ml, EGb761-treatment resulted in a significant decrease in cell viability (*p* = 0.0008, **Fig. 1A**). Therefore, concentration of EGb761 between 25–200 µg/ml was used in the subsequent experiments. This concentration range of EGb761 includes the 100 µg/ml concentration, which was showed to be effective in bEnd.3 cells in a related study [23].

The viability of bEnd.3 cells, pretreated with 25–200 µg/ml EGb761 and then incubated with Aβ_1–42_ oligomer was determined. The concentration of Aβ_1–42_ oligomer (10 µM) was based on the optimization data as described previously [20], [28] with some modifications. The results showed that cells treated with Aβ_1–42_ oligomer alone had significantly reduced viability compared with untreated controls. Pretreatment with EGb761 for 2 h prior to addition of Aβ_1–42_ oligomer resulted in a significant increase in cell viability in a dose-dependent manner from 25 µg/ml to 100 µg/ml EGb761. Fold changes in cell viability following EGb761 and Aβ_1–42_ oligomer treatment, relative to Aβ_1–42_ oligomer alone, were 1.07, 1.19, 1.48 and 1.41-fold at 25, 50, 100 and 200 µg/ml EGb761 respectively (#*p*<0.01, **Fig. 1B**).

### EGb761 prevented Aβ_1-42_ oligomer-triggered apoptosis in bEnd.3 cells

**Figure 2:**
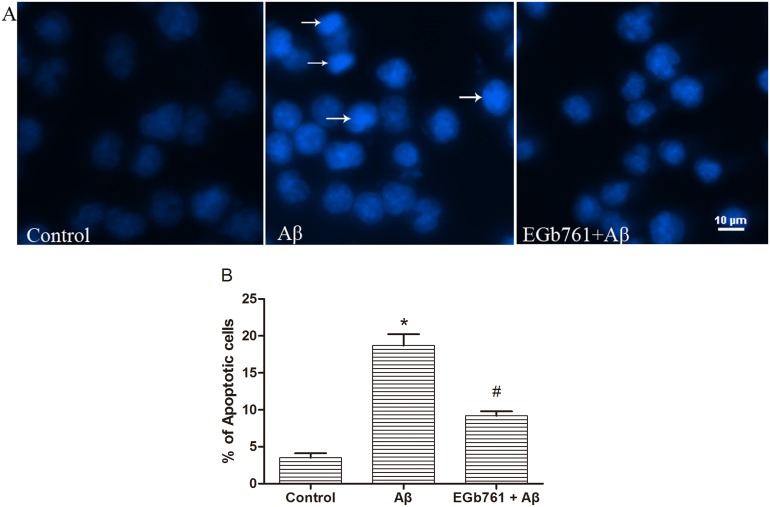
EGb761 prevented Aβ_1__–42_ oligomer-induced apoptosis. bEnd.3 cells were incubated with or without EGb761 (100 µg/mL), followed by incubation with 10 µM Aβ_1–42_ oligomer for 24 h. Cells were then subjected to Hoechst-33258 staining and viewed under a fluorescence microscope. Arrows indicate apoptotic nuclei. The apoptotic nuclei appear shrunken, irregular and fragmented (arrows). Panel B shows the percentage of apoptotic cells. In each group, ten microscopic fields were selected randomly and counted. Results are shown as the Mean±S.E.M. (*p<0.01, Aβ versus Control; #*p*<0.01, EGb761+Aβ versus Aβ).

To investigate the effect of EGb761 on bEnd.3 cell apoptosis, cells were incubated with or without EGb761 for 2 h, followed by treatment with 10 µM Aβ_1–42_ oligomer for another 24 h. We used a concentration of 100 µg/ml EGb761 since this was most effective in the MTT assay (**Fig. 1B**). In the untreated (Control) group, cell nuclei were uniformly stained with the Hoechst-33258 dye (**Fig. 2A** **, Control**), whilst in the group treated with Aβ_1–42_ oligomer alone, bright chromatin condensation and nuclear fragmentation were observed, which is typical of apoptotic nuclei (**Fig. 2A** **, Aβ**). In the EGb761 and Aβ_1–42_ treated group, the nuclei were stained uniformly and the intensity of staining matched the untreated (Control) group (**Fig. 2A** **, EGb761+Aβ**). Apoptotic nuclei were quantitated and the results showed a significant increase in the percentage of apoptotic cells following treatment with Aβ_1–42_ oligomer alone (*p*<0.01, Aβ versus Control, **Fig. 2B**). Treatment with EGb761 prior to addition of Aβ_1–42_ oligomer significantly reduced the percentage of apoptotic cells (*p*<0.01, EGb761+Aβ versus Aβ, **Fig. 2B**).

### EGb761 attenuated Aβ_1-42_ oligomer-induced ROS generation in bEnd.3 cells

**Figure 3:**
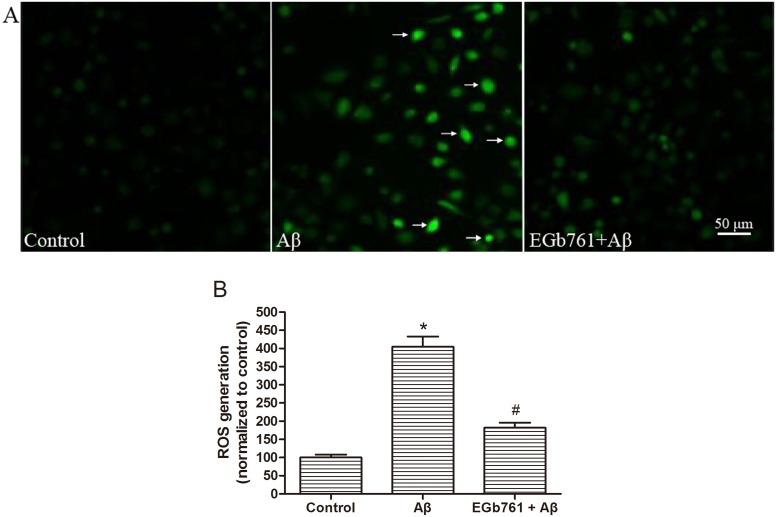
EGb761 attenuated the Aβ_1__–42_ oligomer-induced increase of ROS. Panel A: ROS generation in bEnd.3 cells was evaluated by the oxidation of H_2_DCF-DA to DCF (Fig. 3A, Control) and assessed by inverted fluorescent microscopy (100×). Following treatment for 24 h with 10 µM Aβ_1–42_ oligomer, an increase in fluorescence was detected (Fig. 3A, Aβ). Cells treated with 100 µg/mL EGb761 for 2 h prior Aβ_1–42_ oligomer treatment for 24 h, showed a decrease in fluorescence (Fig. 3A, EGb761+Aβ). Panel B shows the relative levels of intracellular ROS quantified by a microplate reader (488 nm excitation and 525 nm emission), with the results normalized to the control (set at 100). Results are shown as the Mean±S.E.M. (*p<0.01, Aβ versus Control; #*p*<0.01, EGb761+Aβ versus Aβ).

Oxidative stress plays an important role in Aβ-induced cytotoxicity. Therefore, we examined the effect of EGb761 on Aβ_1–42_ oligomer-induced ROS generation in bEnd.3 endothelial cells. A marked increase in ROS generation was detected after treatment with Aβ_1–42_ oligomer alone, with 4.05-fold higher levels of oxidized DCF detected compared with untreated control cells (*p<0.01, Aβ versus Control, **Fig. 3A**). Treatment with EGb761 prior to addition of Aβ_1–42_ oligomer significantly reduced ROS formation induced by the Aβ_1–42_ oligomer (#*p*<0.01, EGb761+Aβ versus Aβ, **Fig. 3A**). These data suggest that EGb761 attenuated Aβ_1–42_ oligomer-induced ROS generation in bEnd.3 cells.

### EGb761 reduced BBB leakage induced by the Aβ_1-42_ oligomer

**Figure 4:**
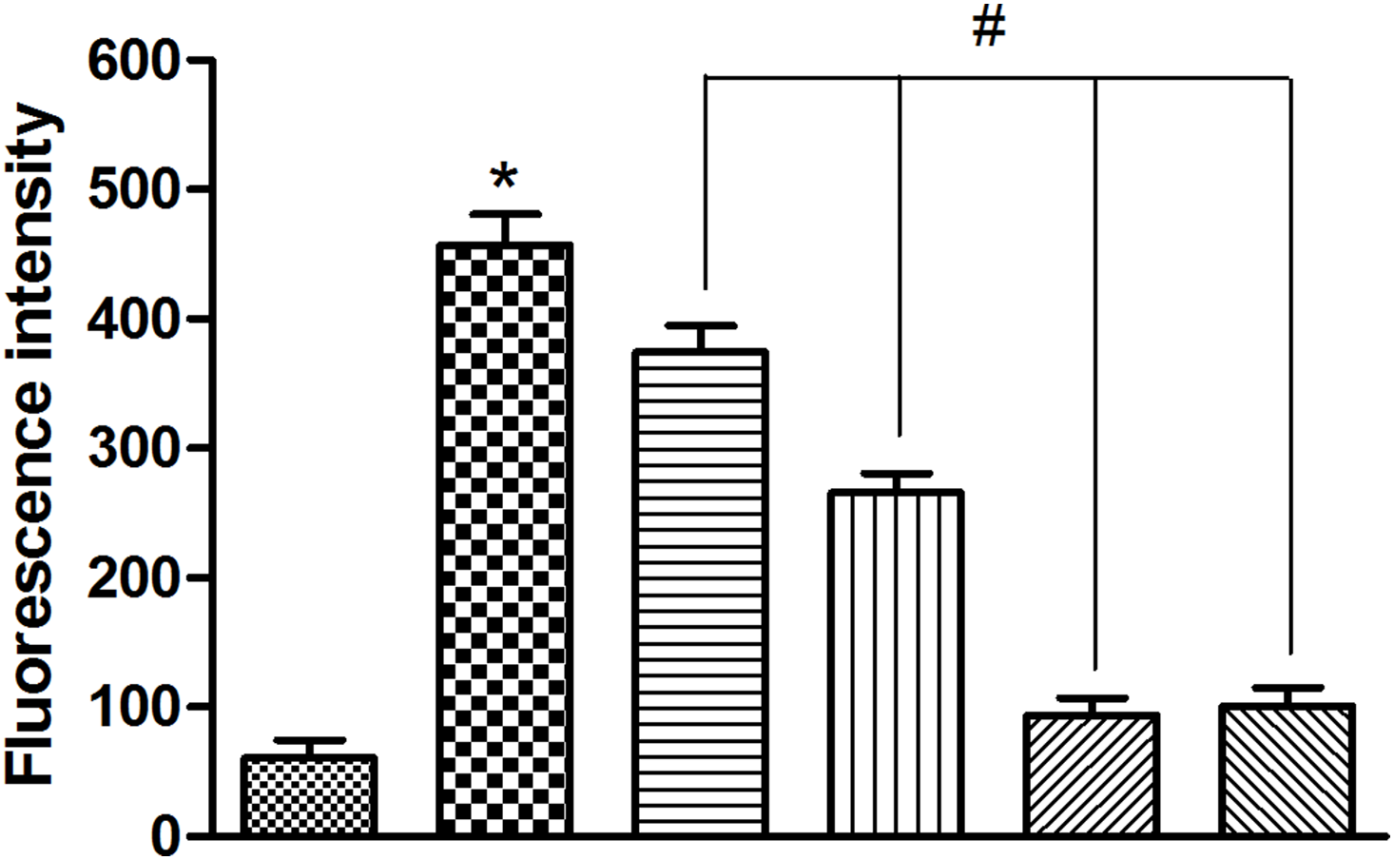
EGb761 decreased BBB permeability in Aβ_1__–42_ oligomer-induced bEnd.3 cells. BBB permeability, evaluated by the Na-F leakage test, was assessed after incubation with 10 µM Aβ_1–42_ oligomer. Cells were incubated with or without various concentrations of EGb761 for 2 h, followed by incubation with Aβ_1–42_ oligomer for 24 h. Then, the absorbance of Na-F was determined by fluorescence spectrophotometry. Results are shown as the Mean±S.E.M. (*p<0.01, Aβ versus Control; #*p*<0.01, EGb761+Aβ versus Aβ).

The BBB is a specialized barrier that controls the transport of various molecules and maintains the integrity of brain by restricting permeability across the brain endothelium [17]. We found that Aβ_1–42_ oligomer increased permeability in cultured bEnd.3 cells (*p<0.01, **Fig. 4**). Pretreatment with EGb761 reversed the barrier permeability damaged induced by Aβ_1–42_ oligomer (#*p*<0.01, **Fig. 4**), and the effect was detected in a dose-dependent manner from 25 µg/ml to 100 µg/ml.

### EGb761 increased protein levels of ZO-1, Claudin-5 and Occludin in Aβ_1-42_ oligomer-induced bEnd.3 cells

**Figure 5:**
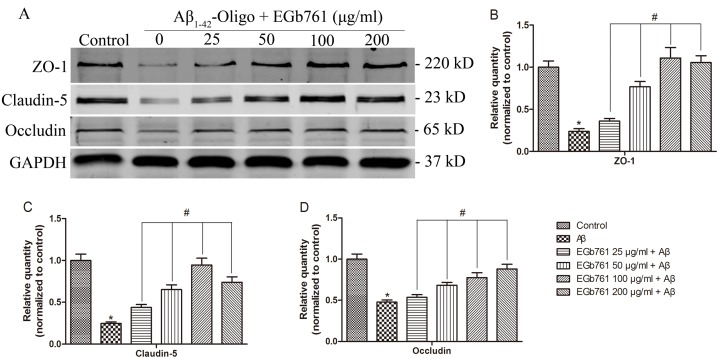
EGb761 increased the expression ZO-1, Claudin-5 and Occludin in Aβ_1__–42_ oligomer-induced bEnd.3 cells. Panel A, cells were incubated with or without various concentrations of EGb761 for 2 h, followed by incubation with Aβ_1–42_ oligomer for 24 h. Then, the expression levels of ZO-1, Claudin-5 and Occludin were determined by Western Blot. Panels B, C and D show the quantitation of the data from Panel A. The Western blots of target proteins were semi-quantitatively analyzed by Image J software and the sum optical density was obtained. Protein levels relative to GAPDH were determined and then normalized to the Control value (i.e. untreated cells), which was set to 1.0. Results are shown as the Mean±S.E.M. (*p<0.01, Aβ versus Control; #*p*<0.01, EGb761+Aβ versus Aβ).

TJs are the most prominent feature of the brain endothelium and are key structures that ensure the integrity of the BBB [28], [29]. On the basis of the above results, we determined the effect of EGb761-pretreatment of bEnd.3 cells on the expression of TJ scaffold proteins ZO-1, Claudin-5 and Occludin. Cells were pretreated with or without EGb761 for 2 h, at concentrations from 25 µg/ml to 200 µg/ml, then exposed to 10 µM Aβ_1–42_ oligomer. Western blot and semi-quantitative analysis showed that the treatment with Aβ_1–42_ oligomer alone significantly decreased the levels of ZO-1, Claudin-5 and Occludin in bEnd.3 cells relative to the control (Ctrl) (*p<0.01, **Fig. 5**). Pretreatment with EGb761significantly increased the levels of those proteins (#*p*<0.01, **Fig. 5**). The protective effect of EGb761 on ZO-1 and Claudin-5 was in a concentration dependent manner from 25 µg/ml to 100 µg/ml, whereas Occludin levels increased in a concentration dependent manner from 25 µg/ml to 200 µg/ml.

### EGb761 reversed Aβ_1-42_ oligomer-induced upregulation of RAGE expression in bEnd.3 cells

**Figure 6:**
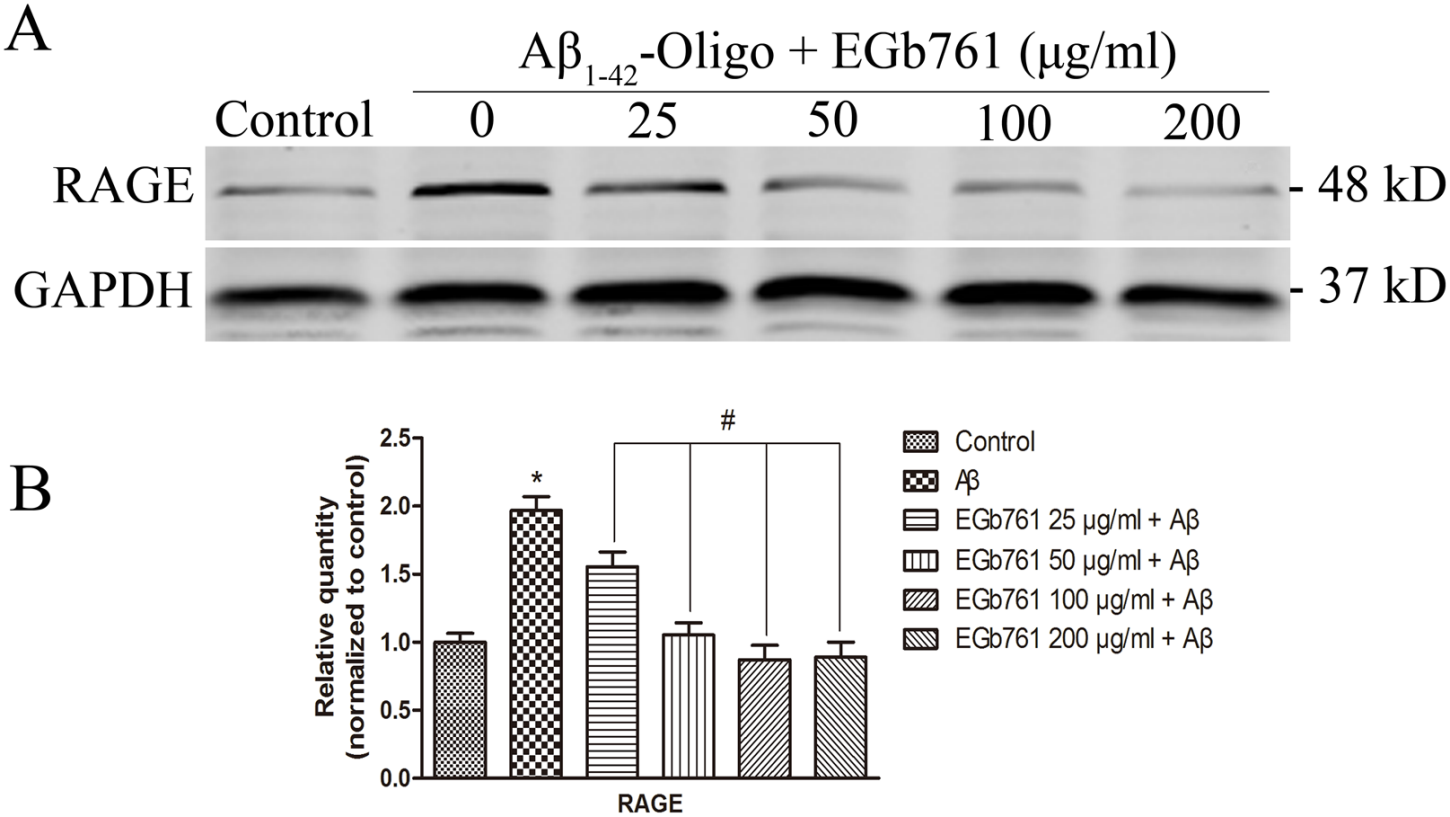
EGb761 reversed Aβ_1__–42_ oligomer-induced up-regulation of RAGE expression in bEnd.3 cells. Panel A, cells were incubated with or without various concentrations of EGb761 for 2 h, followed by incubation with Aβ_1–42_ oligomer for 24 h. Then, RAGE was determined by Western Blot. Panel B is the bar graph based on the result of Panel A. The blots of target proteins were semi-quantitatively analyzed by Image J software and the sum optical density was obtained. Results are shown as the Mean±S.E.M. (*p<0.01, Aβ versus Control; #*p*<0.01, EGb761+Aβ versus Aβ).

In this study, we hypothesized that EGb761 would protect against Aβ-induced BBB disruption through inhibition of RAGE. To test the hypothesis, we determined the effect on the expression of RAGE in Aβ_1–42_ oligomer-induced bEnd.3 cells. Western blot and semi-quantitative analysis revealed that after incubation with Aβ_1–42_ oligomer for 24 h, the expression of RAGE was significantly increased by 1.97-fold when compared with the unexposed Control bEnd.3 cells (*p<0.01, **Fig. 6**). Whereas, treatment of Aβ_1–42_ oligomer-induced bEnd.3 cells with various concentrations of EGb761 led to a significant decrease in the expression of RAGE (#*p*<0.01, **Fig. 6**). Furthermore, the findings suggest that the protective effect of EGb761 on RAGE was in a dose-dependent manner from 25 µg/ml to 100 µg/ml. A further decrease in RAGE expression after pretreated with 200 µg/ml EGb761 was not detectable, when compared with 100 µg/ml EGb761 (**Fig. 6**).

## Discussion

According to the vascular hypothesis of AD, initial vascular damage plays a critical role in the disease development [30]. The origin of BBB dysfunction during AD is not known. However, in a number of AD transgenic animal models, accumulation of Aβ in blood vessels results in the disruption of the BBB [15], [20], [31]. The hypothesis is that BBB breakdown leads to accumulation in the brain of multiple vasculotoxic and neurotoxic macromolecules, and this can initiate functional and structural changes in neurons before Aβ deposition occurs [30]. More importantly, BBB damage impairs vascular clearance of brain Aβ and increases RAGE-mediated influx of blood Aβ into the brain [22], [30]. In this study, we treated cultured immortalized mouse cerebral microvessel endothelial cells with Aβ to model the conditions of the BBB in AD, and subsequently observed the effect of EGb761 on this cell monolayer model of BBB. bEnd.3 cell viability was significantly decreased in response to incubation with Aβ_1–42_ oligomer (**Fig. 1**). There was also a qualitative increase in the number of apoptotic bEnd.3 cells (**Fig. 2**) and an increase in ROS generation (**Fig. 3**). Treatment of EGb761 restored cell viability and reduced both Aβ_1–42_ oligomer-induced cell apoptosis and ROS production *in vitro*.

Intercellular TJs are the most prominent feature of brain endothelium and are responsible for BBB integrity [32]. The physical seal of the BBB is maintained by several different interendothelial TJ complexes that are composed of connecting transmembrane proteins (Occludin and Claudins). These proteins form the primary seal and are linked to accessory cytoplasmic proteins of Zona Occludens family members (ZO-1/2/3 etc), which can also independently link other types of transmembrane proteins to the actin cytoskeleton [33], [34]. Studies have shown that TJ breakdown contributes to the deficiency in BBB function, and abnormal expression of TJ scaffold proteins results in loss of TJ integrity and increased BBB permeability [35], [36]. In this study, we demonstrated that treatment with Aβ_1–42_ oligomer caused significant BBB leakage (**Fig. 4**) and downregulations of ZO-1, Claudin-5 and Occludin (**Fig. 5**). These effects were reduced by EGb761 treatment.

RAGE is a pattern recognition receptor that binds to number of ligands including Aβ [37]. With the exception of the lungs, the basal expression of RAGE is low in physiological conditions but increases with the levels of its ligands [37], [38]. Further, RAGE-ligand interaction and the subsequent up-regulation of RAGE through a positive feedback loop are associated with various diseases, including AD [39]. Accumulating evidence suggests that Aβ plays an essential role in BBB disruption, however, the exact mechanism leading to BBB alteration has not been determined. Recently, Aβ treatment was shown to induce RAGE expression in an *in vitro* study, and furthermore, interaction between Aβ and RAGE triggered an intercellular cascade that disrupted TJ leading to the breakdown of BBB integrity [20], [33]. When pathogenic Aβ species accumulated in the AD brain, either in transgenic models of β-amyloidosis or in the human brain, RAGE expression was increased in affected cerebral vessels, neurons or microglia [40]. This mechanism provides the potential for exacerbating cellular dysfunction due to RAGE-Aβ interactions. The activation of RAGE expressed in neuronal cells promotes synaptic dysfunction and as well leads to neurodegeneration by inducing inflammation in glial cells [9], [41]. Moreover, RAGE-Aβ interaction is implicated in the development of Alzheimer’s neurovascular disorder through various mechanisms. These include mediation of transcytosis of circulating Aβ across the BBB, induction of inflammatory responses in the endothelium, brain endothelial nuclear factor-κB (NF-κB) dependent apoptosis and suppression of cerebral blood flow (CBF), all of which culminate in BBB disruption [19], [42]. In our present study we demonstrated that Aβ_1–42_ oligomer exposure led to a significant increase in the expression level of RAGE in bEnd.3 cells (**Fig. 6**).

Accumulating evidence suggests that RAGE is a potential target for therapies to lower brain Aβ burden, prevent BBB damage, and improve both CBF and behavioral performance [19], [20]. These data suggest RAGE is a potential therapeutic target for AD. A recent study showed that EGb761 markedly reversed the up-regulation of RAGE induced by a CHH condition in a BBB *in vitro* model at both the RAGE mRNA and protein level [23]. These data suggest a rational basis for the therapeutic application of EGb761 in the treatment of AD [23]. Thus, we hypothesized that EGb761 would protect brain ECs against Aβ toxicity via inhibition of RAGE expression. The results indicated that the up-regulation of RAGE expression induced by Aβ_1–42_ oligomer was reversed by treatment with EGb761 (**Fig. 6**).

EGb761 has received a great many attentions because it exerts beneficial effects in conditions which are associated with impaired cognitive function [1], [3], [7]. In the present study, we found that 100 µg/ml of EGb61 showed maximal protection in mainly detection indexes including cell viability, apoptosis, ROS, and the expression levels of ZO-1 and Claudin-5. However, the results also showed that 200 µg/ml of EGb761 resulted in maximal protection with regard to the expression of Occludin. Furthermore, the data indicated that the difference was not significant between 100 µg/ml and 200 µg/ml of EGb761 at the BBB permeability and the expression level of RAGE after incubation with Aβ.

In conclusion, we have presented novel evidence to show that EGb761 effectively prevented Aβ_1–42_ oligomer-induced brain EC damage, which was characterized by reduced cell viability injury, increased cell apoptosis and increased intracellular ROS generation. Furthermore, we found that EGb761 reduced BBB leakage, reversed Aβ_1–42_ oligomer-induced down-regulation of TJ scaffold proteins and prevented the Aβ_1–42_ oligomer-induced up-regulation of RAGE in bEnd.3 cells. To our knowledge, this is the first direct evidence for an effect of EGb761 on brain endothelial cells, and for an effect of EGb761 on the expression of RAGE and TJ scaffold proteins exposed to Aβ_1–42_ oligomer. Our results provide a rational basis for the therapeutic application of EGb761 in the treatment of AD.
